# Is It Possible to Predict Clonal Thrombocytosis in Triple-Negative Patients with Isolated Thrombocytosis Based Only on Clinical or Blood Findings?

**DOI:** 10.3390/jcm10245803

**Published:** 2021-12-11

**Authors:** Tanja Belčič Mikič, Bor Vratanar, Tadej Pajič, Saša Anžej Doma, Nataša Debeljak, Irena Preložnik Zupan, Matjaž Sever, Samo Zver

**Affiliations:** 1 Department of Haematology, University Medical Centre Ljubljana, 1000 Ljubljana, Slovenia; tadej.pajic@kclj.si (T.P.); sasa.anzejdoma@gmail.com (S.A.D.); ipzupan@gmail.com (I.P.Z.); matjaz.sever@kclj.si (M.S.); samo.zver@kclj.si (S.Z.); 2 Department of Internal Medicine, Faculty of Medicine, University of Ljubljana, 1000 Ljubljana, Slovenia; 3 Institute of Biomedical Statistics, Faculty of Medicine, University of Ljubljana, 1000 Ljubljana, Slovenia; bor.vratanar@gmail.com; 4 Clinical Institute for Genomic Medicine, University Medical Centre Ljubljana, 1000 Ljubljana, Slovenia; 5 Department of Clinical Biochemistry, Faculty of Medicine, University of Maribor, 2000 Maribor, Slovenia; 6 Medical Centre for Molecular Biology, Institute of Biochemistry and Molecular Genetics, Faculty of Medicine, University of Ljubljana, 1000 Ljubljana, Slovenia; natasa.debeljak@mf.uni-lj.si

**Keywords:** clonal thrombocytosis, essential thrombocythaemia, calreticulin, platelet count, lactate dehydrogenase

## Abstract

*JAK2*, *MPL,* and *CALR* mutations define clonal thrombocytosis in about 90% of patients with sustained isolated thrombocytosis. In the remainder of patients (triple-negative patients) diagnosing clonal thrombocytosis is especially difficult due to the different underlying conditions and possible inconclusive bone marrow biopsy results. The ability to predict patients with sustained isolated thrombocytosis with a potential clonal origin has a prognostic value and warrants further examination. The aim of our study was to define a non-invasive clinical or blood parameter that could help predict clonal thrombocytosis in triple-negative patients. We studied 237 *JAK2* V617-negative patients who were diagnosed with isolated thrombocytosis and referred to the haematology service. Sixteen routine clinical and blood parameters were included in the logistic regression model which was used to predict the type of thrombocytosis (reactive/clonal). Platelet count and lactate dehydrogenase (LDH) were the only statistically significant predictors of clonal thrombocytosis. The platelet count threshold for the most accurate prediction of clonal or reactive thrombocytosis was 449 × 10^9^/L. Other tested clinical and blood parameters were not statistically significant predictors of clonal thrombocytosis. The level of LDH was significantly higher in *CALR*-positive patients compared to *CALR*-negative patients. We did not identify any new clinical or blood parameters that could distinguish clonal from reactive thrombocytosis. When diagnosing clonal thrombocytosis triple-negative patients are most likely to be misdiagnosed. Treatment in patients with suspected triple negative clonal thrombocytosis should not be delayed if cardiovascular risk factors or pregnancy coexist, even in the absence of firm diagnostic criteria. In those cases the approach “better treat more than less” should be followed.

## 1. Introduction

Thrombocytosis is a common abnormality and a frequent cause of referral to the haematologist. The differential diagnosis of thrombocytosis is broad, including several clonal haematological diseases and many secondary, reactive conditions. In patients with clonal thrombocytosis, the uncontrolled production of platelets is caused by the clonal disease of the haematopoietic stem cell, most commonly seen in essential thrombocythaemia (ET) [1]. Reactive thrombocytosis is most commonly caused by infections, inflammatory diseases, hyposplenism, recent surgery, haemorrhage, malignant disease or most frequently, by iron deficiency [2]. It is of prognostic significance to distinguish between clonal and reactive thrombocytosis as probably only clonal thrombocytosis is related to an increased risk of thrombo-haemorrhagic complications [3]. ET is one of the classical myeloproliferative neoplasms (MPNs) with an increased risk of thrombo-haemorrhagic complications that can occur in more than 20% of patients [4,5]. In a Swedish study performed on 1284 patients with ET, vascular complications were present in up to 35% of those patients [6]. In contrast, patients with reactive thrombocytosis in general do not carry an increased risk of thrombo-haemorrhagic complications [7]. One of the determinants of clonal thrombocytosis, especially in patients with ET, are acquired pathogenic mutations. Three mutations have been identified as driver mutations so far and are known to occur in about 90% of patients with ET. The most frequent mutation is a point mutation in the Janus kinase 2 gene (*JAK2*) V617F [8] that was recognized in 2005 and is nowadays part of a routine molecular genetic testing in patients with suspected ET [9,10,11,12,13]. In 2006, mutations in the thrombopoetin receptor gene (*MPL*) were recognized in *JAK2* V617F-negative patients with ET [14]; the most common are W515L in K [14,15,16]. In 2013, mutations in the calreticulin gene (*CALR*) in *JAK2* V617F- and *MPL*-negative patients with ET were identified [17,18] with the diagnostic value of *CALR* mutations in diagnosing ET confirmed in 2016 [19]. In the remaining 10–15% of patients with ET the molecular genetic background remains unknown. These patients are referred to as triple-negative [20]. Bone marrow examination is also a valuable diagnostic test; however, it is an invasive procedure, can sometimes be inconclusive [2], and may not always be justified, especially in patients with a low risk of thrombotic complications that may not require any treatment. The definitive diagnosis of ET is therefore most difficult in triple-negative patients and mostly relies on inconclusive bone marrow biopsy results.

In triple-negative patients particularly, it would be extremely useful to determine a novel parameter leading to the differentiation between clonal and reactive thrombocytosis. Defining clonal thrombocytosis based on basic clinical or haematological parameters might be especially useful in cases where molecular-diagnostics are not available or the burden of their cost is excessive.

The aim of our study was to analyse patients who were referred to the haematology service at the University Medical Centre Ljubljana for sustained isolated thrombocytosis, and to determine a non-invasive clinical or blood parameter that could predict clonal thrombocytosis, especially in triple-negative patients.

## 2. Methods

### 2.1. Study Design and Study Population

In our study, we included 237 consecutive *JAK2* V617F-negative patients examined at the outpatient haematology clinic who were referred to a haematologist by general practitioners between 7 April 2011, and 13 September 2016. All patients agreed to genetic testing and mostly had a laboratory platelet count-based suspicion of clonal thrombocytosis. The study was retrospective, nonrandomized, and was approved by the Slovenian National Medical Ethics Committee on 14 November 2017 (number 0120-590/2017/5).

We performed molecular-genetic testing in all patients with previously unknown results. The clinical and laboratory data were obtained from hospital records from the date of the first examination in the outpatient clinic. The following parameters were collected: sex, age at the date of the first examination, white blood count (WBC), neutrophil count, erythrocyte count, haemoglobin level, haematocrit level, mean corpuscular volume (MCV), platelet count, ferritin level, iron level, total iron binding capacity (TIBC), saturation of transferrin, C-reactive protein (CRP), lactate dehydrogenase (LDH), the presence of splenomegaly determined by clinical examination, the presence of *MPL* or *CALR* mutations and bone marrow biopsy results.

### 2.2. Blood Count and Biochemical Analysis

Complete blood count was carried out on the Beckman Coulter LH 750 (Beckman Coulter, Brea, CA, USA) or Sysmex XN-1000 (Sysmex Corporation, Kobe, Japan) haematological analysers according to manufacturer’s instructions. General clinical biochemistry and specific protein assays for determination of iron concentration, TIBC, LDH (lactate to pyruvate conversion method), ferritin and CRP levels were performed using ADVIA 1800 or 2400 Clinical Chemistry systems (Siemens Healthineers, Erlangen, Germany). TIBC and serum iron were used to calculate percent saturation of transferrin as (serum iron/TIBC) × 100. All general biochemistry assays and specific proteins assays were carried out according to the manufacturer’s instructions.

### 2.3. Molecular-Genetic Testing

#### 2.3.1. *CALR* Mutation Detection

The detection of *CALR* exon 9 mutations was performed by the fluorescence-based quantitative real-time PCR (qPCR) and post-qPCR analysis with the High Resolution Melting-Curve Analysis (HRM) method, as has been previously published [21,22]. Mutations were defined by type as type 1, type 2, type 1-like, or type 2-like mutations by using AGADIR helix propensity algorithm [23,24].

#### 2.3.2. *MPL* Mutation Detection

The detection of *MPL* NM_005373.2: c.1544G > T, p.(Trp515Leu), or NM_005373.2: c.1543_1544delinsAA, p.(Trp515Lys) mutations was carried out by ipsogen *MPL* W515L/K MutaScreen kit from Qiagen (Germantown, MD, USA) according to manufacturer’s instruction. The duplicate samples of 25 ng of purified granulocytes DNA were used for the fluorescence-based qPCR and allelic discrimination analysis. The analysis was performed on the ABI PRISM ViiA 7 Real-Time PCR system (Applied Biosystems—ThermoFisher Scientific, Waltham, MA, USA).

### 2.4. Patient Evaluation

Based on clinical examination, laboratory data, the presence of *CALR* or *MPL* mutations, bone marrow examination results and follow-up data, patients were retrospectively diagnosed by a panel of three expert haematologist with either clonal or reactive thrombocytosis.

### 2.5. Statistical Analysis

#### 2.5.1. Descriptive Statistics

First, we examined data for missing values. The following variables had missing data: ferritin (1.3%), iron level (1.3%), TIBC (2.2%), transferrin saturation (2.2%), CRP (0.9%) and LDH (0.4%). We merged patients with type 1 *CALR* mutation, type 2 *CALR* mutation, and type 2-like *CALR* mutation into one category (*CALR*-positive patients). One patient (0.4%) who was diagnosed with a missense *CALR* mutation was considered as having a missing value on this variable. We calculated descriptive statistics for blood parameters for clonal and reactive thrombocytosis. We calculated median values and associated interquartile range for numerical blood parameters with skewed distributions.

#### 2.5.2. Predictive Value of Blood Parameters and Gene Expression

The main goal of our study was to determine the predictive value of blood parameters in relation to the cause of thrombocytosis (clonal or reactive) as a response variable. The relationship between the cause of thrombocytosis and blood parameters was analysed using logistic regression. Based on previous studies and the diagnostic criteria for MPN we first considered platelet count and LDH as predictor variables. Because of the non-linear effect of platelet count, we introduced restricted cubic splines in the model to model the non-linear relationship between log-odds and platelet count. We placed three knots in the logistic model. The location of the knots was determined based on the suggested quantile values in the literature [25]. In model A, we included platelet count and LDH as predictor variables and estimated parameters on the whole study population. In model B, we classified *CALR*-positive and *MPL*-positive patients as having clonal thrombocytosis and estimated parameters of the logistic model on a subsample of triple-negative patients. Later, we examined whether we could improve either model by including other blood parameters in the model. We additionally included the following blood parameters in each model: WBC count, neutrophil count, erythrocyte count, haemoglobin level, haematocrit level, MCV, ferritin level, iron level, TIBC, saturation of transferrin, CRP, and the presence of splenomegaly as well as gender and age of diagnosis. We did not include *CALR* or *MPL* mutations in the logistic model because positive gene expression is a deterministic indicator of clonal thrombocytosis. We included the same predictor variables in model B as in model A. We estimated test error using leave-one-out cross-validation. Lastly, we also depicted the diagnostic ability of the platelet count with a receiver operating characteristic (ROC) curve. To test the mean difference in LDH between *CALR*-negative and *CALR*-positive patients on a subsample of patients with primary thrombocytosis we used Welch’s t-test for independent samples. Statistical analysis was performed with R version 3.5.1 [26]. The significance level was set at α = 0.05 for all statistical tests.

## 3. Results

### 3.1. Molecular-Genetic Testing and Haematological Diagnosis

Among 237 *JAK2* V617F-negative patients examined at the outpatient clinic we excluded six patients with an unsuitable DNA sample for the genetic analysis so our final group included 231 patients. Twenty-two patients (9.5%) were diagnosed with *CALR* mutations: fourteen patients (6.1%) with type 1 mutation, two patients (0.86%) with type 2 mutation, five patients (2.16%) with type 2-like mutations, and one patient (0.43%) was diagnosed with a missense mutation. One patient (0.43%) was diagnosed with an *MPL* mutation.

Considering all the available clinical, haematological, molecular-genetic and bone marrow findings the panel of three expert haematologists diagnosed fifty-one patients (22.1%) with clonal thrombocytosis and 180 patients (77.9%) with reactive thrombocytosis. The main characteristics of our patients are presented in Table 1. Patients with clonal thrombocytosis in our sample had much higher median values of platelets, ferritin, TIBC, and LDH compared to patients with reactive thrombocytosis. In 148 patients (64%) the level of platelets was lower than 450 × 10^9^/L. Bone marrow biopsies were performed in 41 patients (17.7%), in nine patients (3.9%) the results confirmed ET, in six patients (2.6%) primary myelofibrosis, in three patients (1.3%) possible other haematological diseases and in 23 patients (9.9%) the results of bone marrow biopsies were either inconclusive or negative. Patients that were diagnosed with reactive thrombocytosis were followed-up with their general practitioners. In case of persistence or recurrence of thrombocytosis or the development of thrombo-haemorrhagic complications the patients were referred back to the haematologist. The follow-up period with the general practitioners was from three to eight years. During the study period no patients that were diagnosed with reactive thrombocytosis by the haematologist were referred back to the haematology service due to persistence or recurrence of thrombocytosis or thrombo-haemorrhagic complications, suggesting that no patients were misdiagnosed with reactive thrombocytosis.

### 3.2. Prediction of Clonal Thrombocytosis Based on Blood Parameters

#### 3.2.1. Model A

One patient was excluded from the analysis due to a missing LDH value. The results show that model A was statistically significant ($\chi^{2}\left(3\right)=81.8,p<0.001$). All predictor variables in the model A were statistically significant (Table 2). The estimated coefficient for LDH had a positive value meaning that patients with higher values of LDH had a higher probability of being diagnosed with clonal thrombocytosis. Next, the results suggest that patients with higher platelet count had a higher probability of being diagnosed with clonal thrombocytosis. However, the relationship between log-odds and platelet count was not linear. Based on estimated parameters we can see that the relationship between log-odds and platelets was very strong at low counts of platelets but was weaker at higher counts of platelets. Test error for the model A was low (test error = 15.7%, specificity = 93.9%, sensitivity = 51%).

#### 3.2.2. Model B

Two patients were excluded from the analysis, one patient with a *CALR* missense mutation and one patient with a missing LDH value, respectively. The results show that the logistic regression model was statistically significant ($\chi^{2}\left(3\right)=35.7,p<0.001$). We can again observe the non-linear effect of platelet count on the log-odds (Table 2). However, in contrast to model A, LDH was not a statistically significant predictor variable in this model. For model B we estimated test error in the following way: first, we correctly classified *CALR* and *MPL* positive patients with clonal thrombocytosis and then estimated probabilities based on the second model for the remaining patients (triple-negative). Test error for the model B was lower compared to the estimated test error in model A (test error = 12.7%, specificity = 98.3%, sensitivity = 49%).

**Figure 1:**
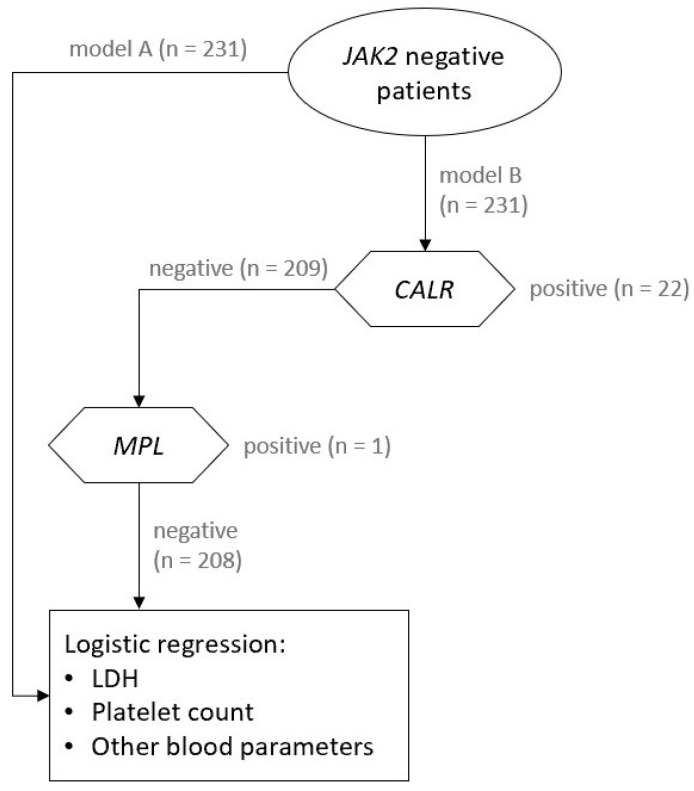
The schematic presentation of our statistical analysis with models A and B.

In addition, we examined whether we could improve both models by including other blood parameters listed in Table 1 as predictor variables. To test this hypothesis, we included other blood parameters listed in Table 1 in the model A and B and fitted the logistic regression model using backward elimination. However, among new predictor variables, none remained in the model A or B after the backward elimination procedure. The rest of the predictors did not improve either model statistically significantly, therefore we did not include them in our final model. The schematic presentation of approach to both models is presented in Figure 1.

### 3.3. Diagnostic Value of Platelet Count

**Figure 2:**
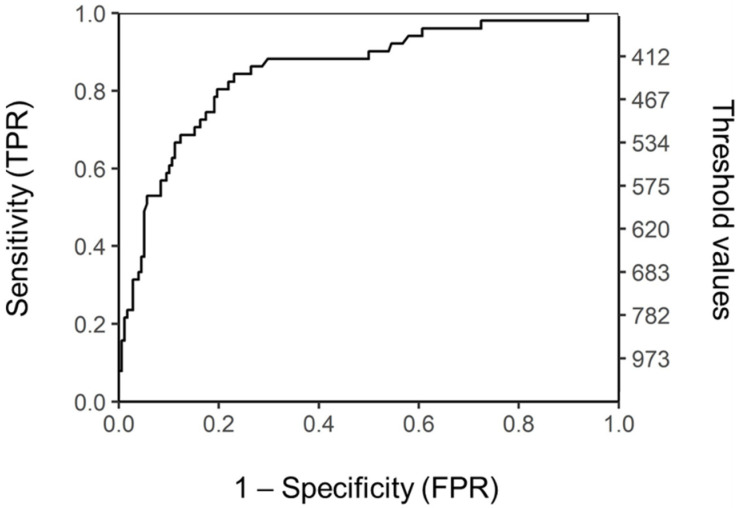
ROC curve for platelets as binary classificatory and thrombocytosis as a class variable. Threshold values are presented on the right axis (scale is not linear).

Because platelet count seemed to be the most important predictor variable in both models we also inspected the diagnostic value of platelets as a binary classificatory. We plotted the ROC curve with platelets as a classificatory and thrombocytosis as a class variable (see Figure 2). The optimal point (Youden’s index) was identified at the value of 449 × 10^9^/L.

### 3.4. The Value of LDH

In addition, we compared LDH mean values between *CALR*-positive and triple-negative patients on a subsample of patients with clonal thrombocytosis. The explanatory analysis revealed that the difference between the means was statistically significant, t (27.4) = 3.45, *p* = 0.002. Patients with *CALR*-positive mutation had higher values on LDH (M = 4.92, SD = 2.16) than patients who were triple-negative (M = 3.16, SD = 1.09).

## 4. Discussion

To our knowledge, this is the first study reporting on a search for a novel clinical and/or laboratory non-invasive marker that could distinguish between clonal and reactive thrombocytosis. This is of great importance for regular clinical work, especially in some groups of patients such as pregnant women.

We included only *JAK2*-negative patients as this mutation is the most common in patients with clonal thrombocytosis and has been part of a routine molecular-genetic testing in all patients with suspected clonal thrombocytosis at the time of examination. The downside of our study is the fact that the diagnosis of clonal versus reactive thrombocytosis was established retrospectively and therefore it was not in all cases based on the current World Health Organisation (WHO) diagnostic criteria. It also took several weeks or months from patients’ referral from general practitioner to a haematologist. In many cases (i.e., inflammation, iron deficiency) reactive causes of thrombocytosis had already subsided resulting in the absence of significant thrombocytosis. In extension, the diagnosis of either clonal or reactive thrombocytosis was established by a panel of three experienced haematology consultants in accordance with real life national guidelines valid at our department. At the time of the study none of the patients that were diagnosed with reactive thrombocytosis were referred back to the haematologist suggesting that no patients were misdiagnosed.

As proposed from the literature, elevated inflammatory markers such as C-reactive protein [27] and iron deficiency markers such as iron saturation, low ferritin and low serum iron concentration [28] are related to reactive thrombocytosis. Our aim was to determine whether we could distinguish between clonal and reactive thrombocytosis based on such common blood parameters. We applied two predictive models: model A was applied to all patients regardless of their molecular-genetic status and model B only on the subpopulation of triple-negative patients. The aim of model A was to distinguish between clonal and reactive thrombocytosis not having to use driver mutations which could prevent excessive diagnostic workup costs. Additionally, avoiding molecular-genetic testing could lessen the patients’ psychological burden of genetic testing. In model A the only statistically significant predictors of clonal thrombocytosis were platelet count and LDH. The number of platelets was an important predictor with the absolute number of platelets at 449 × 10^9^/L showing the most exact differentiation between clonal and reactive thrombocytosis. Our study shows that the threshold for clonal thrombocytosis is 449 × 10^9^/L which is consistent with the WHO criteria [19] which require the persistence of thrombocytosis with platelet count at or above 450 × 10^9^/L. In higher levels of platelets, however, the platelet count itself is a less important predictor of clonal or reactive thrombocytosis. This is consistent with findings from the literature that show that very high levels of platelets can also be observed in patients with tumours or inflammatory diseases [29].

Although rather nonspecific, LDH is known to be increased in patients with ET [30,31], with a confirmed prognostic value [32]. LDH may also be a sensitive marker of occult pre-PMF [32], which has only recently been recognized as a separate entity [19] in patients with previously diagnosed WHO-defined ET. Pre-PMF represents a form of clonal thrombocytosis similar to ET with the main distinction being the prognosis of the disease. A study by Mudireddy et al. showed there was a direct correlation between increased serum LDH, leucocytosis, increased platelet count, and the presence of splenomegaly which all are markers of a haematological disease [32]. LDH is therefore a logical predictor of clonal thrombocytosis. Other parameters presented in Table 1 which were included in model A were not statistically significant predictive factors in distinguishing clonal from reactive thrombocytosis. The aetiology of reactive thrombocytosis is diverse and often multifactorial which might explain why no other single marker was found to be statistically significant in the model.

In model B performed on triple-negative patients the most important predictor of clonal thrombocytosis was platelet count. LDH, in contrast, was not a statistically significant predictor of clonal thrombocytosis. One explanation is that the test power was much lower on the subsample of triple-negative patients. It is also true that *CALR* mutation might partially explain the effect of LDH on the type of thrombocytosis as *CALR*-positive patients in our study had higher values of LDH compared to *CALR*-negative patients. *CALR*-positive patients given their positive molecular genetic marker were more likely to be accurately diagnosed with clonal thrombocytosis compared to *CALR*-negative patients. Again, in model B there was no other statistically significant prediction marker of clonal thrombocytosis.

Our study shows that despite molecular genetic advances in recent years the accurate diagnosis of ET based on patient’s history, clinical examination, and basic laboratory parameters remains difficult, especially in triple-negative patients. It is most accurate that patients with increased sustained isolated thrombocytosis with the absolute number of platelets above 449 × 10^9^/L or less do not have clonal thrombocytosis. Also, LDH is a statistically significant marker of myeloproliferation making the diagnosis of clonal thrombocytosis easier. Other blood and clinical tests that are usually performed in patients with suspected clonal thrombocytosis were, unfortunately, not statistically significant predictors of clonal thrombocytosis. As patients with clonal thrombocytosis, especially ET, are more prone towards thrombotic complications it is especially important to distinguish between clonal and reactive thrombocytosis. As this seems to be difficult in triple-negative patients these patients should be considered as having clonal thrombocytosis. In our opinion they should be treated according to their thrombotic risk especially if the increased platelet count is sustained and no reactive cause was found or treated. The standard therapy in most patients is acetylsalicylic acid which has acceptable side effects that do not outweigh the potential benefit of treatment.

We also hypothesized that the number of patients with suspected ET was overestimated. More than three quarters (77.9%) of patients were diagnosed with reactive thrombocytosis which is in accordance with our prediction and was also determined in several similar studies [3,33,34,35,36]. Furthermore, our study confirms that most referrals to the haematologist with suspicion of ET are not necessary. The approach to patients with isolated thrombocytosis in primary care should therefore be improved.

The most common cause of thrombocytosis in our study was iron deficiency, that was present in almost half of patients with reactive thrombocytosis. Iron deficiency is one of the most common causes of reactive thrombocytosis [2]. In case of severe iron deficiency extreme thrombocytosis may be present (platelet count above 1000 × 10^9^/L) [37]. Although there is no correlation between ferritin levels and platelet count [38,39,40], studies have shown that iron-depleted individuals have higher platelet counts [41]. Iron seems to have an inhibitory role on platelet production as iron supplements reduce platelet count [42]. Also, megakaryopoiesis in iron deficiency state changes [43]. That is the reason it is important to exclude iron deficiency in all patients with thrombocytosis as it may be present even in the absence of anaemia.

In our study, reactive thrombocytosis was related to infectious diseases and inflammatory conditions in 42% of patients. Inflammatory conditions are a common cause of thrombocytosis and are related to an elevated C-reactive protein or accelerated sedimentation rate [29] as well as elevated pro-inflammatory cytokines such as interleukin-6 (IL-6) and interleukin-11 (IL-11) [44]. In our study, it occurred in patients with rheumatoid arthritis, gout, and immune-mediated eye disease. In patients with rheumatoid arthritis pro-inflammatory cytokines that are related to the disease itself also stimulate megakaryopoiesis and thrombopoiesis. Thrombocytosis in patients with rheumatoid arthritis can be an indicator of disease activity [45], in patients with ulcerous colitis the number of platelets may be used as a prognostic indicator of disease relapse [46].

Thrombocytosis can also occur in patients with various infectious conditions [47] but is usually transient and resolves in one to two days of antibiotic therapy or surgical removal of the infectious cause [34].

Thrombocytosis in our study was reported after surgical procedures in 8% of patients with reactive thrombocytosis. Tissue injury which appears after surgical procedures can stimulate the production of platelets via inflammatory mediators such as IL-6. This type of thrombocytosis can persist up to six weeks after tissue injury with the highest platelet count in the first two weeks after tissue injury [48].

In 6% of patients in our study reactive thrombocytosis occurred after splenectomy. As generally known, thrombocytosis can be present in up to 75% of patients after splenectomy [49]. As the number of all circulatory platelets increases [50], thrombotic complications are possible, including portal vein thrombosis [51]. It is not entirely clear if thrombocytosis after splenectomy can be related to increased risk of portal vein thrombosis as it does not occur in all patients with thrombocytosis after splenectomy. In addition, thrombocytosis is not present in all patients with portal vein thrombosis after splenectomy [52]. According to a large European study, thrombocytosis after splenectomy is not related to an increased risk of thrombotic complications [3].

Our study confirms that the most common causes of reactive thrombocytosis should be excluded before referral to the haematologist. Platelet count should be analysed at least at two occasions.

Moreover, as ET is more common in women than in men and an estimated 20% of patients are younger than 40 years of age [53] a non-negligible group of patients with ET are pregnant women. During pregnancy, there is an increased risk for maternal complications such as venous thromboembolism, major bleeding events, and preeclampsia as well as foetal complications, for example, foetal loss, preterm delivery, and intra-uterine growth restriction [54,55,56]. Thrombocytosis, already present before pregnancy with no other cause, can be a sign of clonal hematologic disease. In the case of ET, platelet count during pregnancy may even be normal as gestational thrombocytopenia may contribute to the pseudo-normalisation of platelet count. Due to serious potential complications, pregnant women with thrombocytosis should in our opinion be carefully monitored and treated as having ET even if the diagnosis cannot be confirmed with non-invasive tests.

We can conclude that the diagnosis of clonal thrombocytosis is especially difficult in triple-negative patients. In this group of patients, it would be extremely beneficial to find a novel marker that would lead to the diagnosis of ET. Among molecular-genetic markers, next generation sequencing (NGS) shows the discovery of novel mutations in patients with MPN, some of them with a prognostic value [57]. Some of these are mutations in the following genes *LNK, TET2, DNMT3A, IDH1/2, CBL, ASXL1,* and atypical *JAK2* (JAK2V626F in JAK2F556V) and *MPL* mutations (MPLS204P in MPLY591N, S204P) [58,59,60]. Some of the laboratories around the world are already using NGS as a diagnostic tool in patients with suspected ET which could in the future represent a model of personalized medicine [61,62]. It is also possible other laboratory markers that have so far been defined as nonspecific will become important in distinguishing clonal from reactive thrombocytosis.

## 5. Conclusions

Platelet count above 449 × 10^9^/L and increased level of LDH are suspicious for clonal thrombocytosis. The diagnosis of clonal thrombocytosis in triple-negative patients cannot be firmly made based on non-invasive clinical or laboratory findings. In triple-negative patients with sustained isolated thrombocytosis clonal thrombocytosis should be highly suspected. Appropriate treatment in order to prevent potential thrombo-haemorrhagic complications should not be delayed. The approach “better treat more than less” should be followed.

## Figures and Tables

**Table 1:** Main characteristics of our patients (*n* = 231).

Characteristics	Clonal Thrombocytosis (*n* = 51)	Reactive Thrombocytosis (*n* = 180)
Female, *n* (%)	34 (67)	139 (77)
Age at onset, mean (SD), years	57.5 (18.5)	48.9 (16.3)
Erythrocyte count, median (Q1–Q3), ×10^12^/L	4.38 (4.17–4.66)	4.51 (4.3–4.8)
Haemoglobin, median (Q1–Q3), g/L	130 (112.5–139)	130 (122–140.2)
Haematocrit, median (Q1–Q3)	0.39 (0.36–0.42)	0.39 (0.37–0.41)
MCV, median (Q1–Q3), fl	88.4 (85.3–91.7)	86.4 (83.4–89.4)
Platelet count, median (Q1–Q3), ×10^9^/L	605 (473–726)	403 (361–444)
WBC count, median (Q1–Q3), ×10^9^/L	9.2 (7.9–11.1)	8.61 (6.97–10.66)
Neutrophil count, median (Q1–Q3), ×10^9^/L	6 (4.62–7.21)	5.19 (4.15–7.14)
Ferritin, median (Q1–Q3), µg/L	102 (32.5–189.5)	36.5 (12.2–90.5)
Saturation of transferrin, median (Q1–Q3), %	23 (14.5–32)	19.3 (11.9–31)
Iron, median (Q1–Q3), µmol/L	13.1 (9.5–17.35)	12 (7.45–18.2)
TIBC, median (Q1–Q3), µmol/L	54.6 (50.3–66.5)	61.5 (55.1–68.5)
LDH, median (Q1–Q3), µkat/L	3.22 (2.82–4.25)	2.81 (2.5–3.13)
C-reactive protein ≥ 5, mg/L	17 (34)	62 (35)
Splenomegaly, *n* (%)	3 (6)	2 (1)
*CALR*-positive patients, *n* (%)	22 (43)	-
*MPL*-positive patients, *n* (%)	1 (1.96)	-
Triple negative patients, *n* (%)	28 (54.9)	-

MCV, mean corpuscular volume; WBC, white blood cell; TIBC, transferrin iron binding capacity; LDH, lactate dehydrogenase.

**Table 2:** Parameter estimates, odds ratio (OR) and associated inferential statistics for all models.

Predictor	Coefficient	SE	*p*	OR
Model A				
Platelet count	/	/	<0.001 **	/
Linear effect	0.025	0.007	<0.001 **	1.025
Non-linear effect	−0.028	0.01	0.006 *	0.973
LDH	0.518	0.17	0.002 *	1.679
Model B				
Platelet count	/	/	<0.001 **	/
Linear effect	0.033	0.011	0.003 *	1.034
Non-linear effect	−0.03	0.012	0.01 *	0.97
LDH	0.017	0.231	0.941	1.017

Note. Thrombocytosis (response variable): 0 = reactive thrombocytosis, 1 = clonal thrombocytosis. Model A: *n* = 230. Model B: *n* = 207 (*CALR* and *MPL*-negative patients). Missing cases were excluded list wise for each model. OR = odds ratio. * *p* < 0.05, ** *p* < 0.001.

## Data Availability

The data supporting this work is available from the corresponding author on reasonable request.

## Abbreviations

CALR	calreticulin
CRP	C-reactive protein
ET	Essential thrombocythaemia
HRM	High resolution melting
IL-6	Interleukin-6
IL-11	Interleukin-11
JAK-2	Janus kinase-2
LDH	Lactate dehydrogenase
MCV	Mean corpuscular volume
MPL	Thrombopoetin receptor
MPN	Myeloproliferative neoplasm
NGS	Next-generation sequencing
OR	Odds ratio
PMF	Primary myelofibrosis
PV	Polycythaemia vera
Q1–Q3	Interquartile range
qPCR	Quantitative polymerase chain reaction
ROC	Receiver operating characteristic
SD	Standard deviation
TIBC	Total iron binding capacity
WBC	White blood count
WHO	World Health Organisation
